# Supplementary material for: Do people have an ethical obligation to share their health information? Comparing narratives of altruism and health information sharing in a nationally representative sample
Source: PLoS One. 2020 Dec 31;15(12):e0244767. doi: 10.1371/journal.pone.0244767 (PMC7774955; doi:10.1371/journal.pone.0244767)
Supplement: S1 File — (PDF) [file pone.0244767.s003.pdf]

Deleted:

NORC at the UNIVERSITY of CHICAGO

AmeriSpeak

**Client**	University of Michigan
**Project Name**	Longitudinal Survey of Epidemiology
**Project Number**	8478
**Survey length (median)**	20 minute survey
**Population**	21+ gen pop
	African American/Hispanic and <=200% FPL oversamples
**Pretest**	N=100
**Main**	N=2000
**MODE**	Web only
**Language**	English
**Incentive**	5,000 points
**Survey description**	Healthcare issues
**Eligibility Rate**	100%

Deleted:

Standard demographic preloads:

Var Name	Var Type	Var length	Variable Label
S_AGE	Numeric	5	Age
S_GENDER	String	8	Gender
S_RACETH	Numeric	8	Race/ethnicity
S_EDUC	Numeric	6	Education
S_MARITAL	Numeric	9	Marital Status
S_EMPLOY	Numeric	8	Current employment status
S_INCOME	Numeric	8	Household income
S_STATE	String	7	State
S_METRO	Numeric	7	Metropolitan area flag
S_INTERNET	Numeric	10	Household internet access
S_HOUSING	Numeric	9	Home ownership
S_HOME_TYPE	Numeric	11	Building type of panelist's residence
S_PHONESERVC	Numeric	11	Telephone service for the household
S_HHSIZE	Numeric	8	Household size (including children)
S_HH01	Numeric	6	Number of HH members age 0-1
S_HH25	Numeric	6	Number of HH members age 2-5
S_HH612	Numeric	7	Number of HH members age 6-12
S_HH1317	Numeric	8	Number of HH members age 13-17
S_HH18OV	Numeric	8	Number of HH members age 18+
S_file_date	Date	11	
S_GENFRACE	Numeric	8	GenF custom race

These populated as a pre-load when the panelists get sampled into the survey

Standard sample preloads

Variable Name	Variable Type	Variable Label
Username	Numeric	Analogous to Member_PIN
P_Batch	Numeric	Batch Number (if only one assignment, then everyone will be 1)
Dialmode	Numeric	CATI Dialmode (predictive, preview, etc)
P_LCS	Numeric	Life cycle stage, 0=released but not touched
Y_FCELLP	String	
Surveylength	Numeric	Estimated length of survey
SurveyId	Numeric	Survey ID# in A4S
Incentwcomma	String	Study specific
P_Hold01	Numeric	Prevents dialing cases without phone numbers

Custom survey-specific preloads

Variable Name	Variable Type	Variable Label
P_PARTYID7	Numeric	1 "Strong Democrat"<br>2 "Moderate Democrat"<br>3 "Lean Democrat"<br>4 "Don't Lean/Independent/None"<br>5 "Lean Republican"

		6 "Moderate Republican"<br>7 "Strong Republican"<br>***only preload responses IF NOT MISSING PARTYID7**
FPL200	Numeric	1 “200FPL”<br>0 “Not 200FPL”

Deleted:

This survey will use the following RND_xx variables:
Note, these are randomized in the script (NOT preloads)

**RND_xx**	**Associated survey Qs**
RND_00	
RND_01	
RND_02	
RND_03	
RND_04	
RND_05	
RND_06	

Deleted:

Please include the following options for all questions in CATI:
77 DON'T KNOW
99 REFUSED

Please code refusals in CAWI:
98 IMPLICIT REFUSAL, WEB SKIP
Do not code 77 Don't Know/99 Refused options in CAWI unless written in item response options

---

Text shown in green includes researcher notes and should not be included in the programming.

[START OF SURVEY]

CREATE DATA-ONLY VARIABLE: QUAL
1=Qualified Complete
2=Not Qualified
3=In progress

AT START OF SURVEY COMPUTE QUAL=3 "IN PROGRESS"

CREATE MODE_START
1=CATI
2=CAWI

HOVER TEXT PROGRAMMING: BELOW IS THE HOVER TEXT THAT SHOULD BE DISPLAYED WHERE INDICATED THROUGHOUT THE SURVEY

HT_1: My healthcare system
"Your healthcare system" refers to the healthcare professionals and institutions that you personally interact with when getting health care.

HT_2: The healthcare system
"The healthcare system" refers generally to the healthcare system in this country.

HT_3: Healthcare providers
Health care providers include people such as doctors and nurses who provide medical treatment.

HT_4: Electronic health record
A digital version of your paper chart or medical record. An electronic health record contains your medical and treatment history including diagnoses, medications, treatment plans, immunization dates, allergies, radiology images, and laboratory and test results.

HT_5: Health information

Health information includes information about you and your medical treatment history including diagnoses, medications, treatment plans, immunization dates, allergies, radiology images, and laboratory and test results.

HT_7: De-identified [health information or biospecimens]
De-identified means that “identifying information” about *you* is *removed* from your health information. Identifying information includes things like your name, address, date of birth, etc.

HT_8: University researcher

A university researcher is a person who works for colleges or universities. University researchers might use health information to understand how people use the healthcare system, how healthcare providers treat patients, and a wide variety of other health related topics. University researchers might also use biospecimens for research on how illness works, which treatments are most effective, or how genetics affect illness. University researchers may or may not be connected to your hospital in some way.

HT_9: Biospecimens
Biospecimens include blood from a blood test, or tissue or tumor samples from a biopsy. Your biospecimens contain your DNA.

HT_10: Identified [health information or biospecimens]
Identifying information includes things like your name, address, date of birth, etc. Identified biospecimens are biospecimens that include identifying information about you.

HT_11: Quality analysts

Quality analysts are people who work for hospitals or clinics. They use patient health information at their hospital or clinic to check on, and improve, how their organization is working. They often study the cost of healthcare, their organization’s efficiency on things like waiting room times, and the health of patients at their hospital or clinic.

HT_12: Commercial companies

Commercial companies are third-party companies that are not part of a hospital. For example, a third-party commercial company may conduct genetic tests and analyze information for a hospital or healthcare provider for a fee when a hospital is not be able to conduct the test on their own.

(Project name) Draft
Date: (Quex start date)

[DISPLAY – WINTRO_1]
Thank you for agreeing to participate in our new AmeriSpeak survey! To thank you for sharing your opinions, we will give you a reward of [INCENTWCOMMA] AmeriPoints after completing the survey. As always, your answers are confidential.

Please use the "Continue" and "Previous" buttons to navigate between the questions within the questionnaire. Do not use your browser buttons.

Deleted:

[DISPLAY_1]
This is a survey about your healthcare experience and your opinions about how [HT_5] health information is used and shared.

[SPACE]

The survey includes a short video, which <u>needs to be viewed with sound</u>. If you are not able to have your sound on at this time, feel free to take this survey later when you can. Alternatively, if you will not be able to view the video with sound, we have a transcript of the video that can be read instead.

[DISPLAY_2]

We all use [HT_2] the healthcare system or know people who do. This system includes the healthcare provider, like a doctor or nurse, who you visit when you're sick or for routine visits. It also includes hospitals and people who work on quality improvement, and administrators who make decisions about how clinics and hospitals are run.

[SPACE]

When we ask about <i>"*[HT_2] the healthcare system*"</i> we are asking generally about [HT_2] the healthcare system in this country. When we ask about "*your* healthcare system" we are asking about the healthcare professionals and institutions that you personally interact with.

[SPACE]

We are interested in your thoughts and ideas. There are no "right" or "wrong" answers.

[GRID; SP]

Q1.

Please state if the following are true or false:

GRID ITEMS [RANDOMIZE]:

A. My [HT_3] healthcare provider uses an [HT_4] electronic health record
B. I have used a patient portal to access my [HT_5] health information online
C. I am worried about being able to pay medical bills
D. I am confident my health insurance covers my medical needs

RESPONSE OPTIONS:

1. True
2. False
77. Not sure

PM: PLEASE MAKE SURE THE DATE TIME RULE ALWAYS FOLLOWS FIRST QUESTION
INSERT ITEM TIMESTAMPS: TIME_FIRST, DATE_FIRST

Deleted:

THE REST OF THE CLIENT INSTRUMENT GOES HERE.

[SHOW IF Q1_2=1]

[SP]

Q1A1

I have more than one patient portal.

RESPONSE OPTIONS:

1. Yes
2. No

[RANDOMIZE RESPONSE OPTIONS]

[MP]

Q2.

In the past 12 months, have you...
[SPACE]
<unbold><i>*Please select all that apply.*<unbold></i>

RESPONSE OPTIONS:

1. …seen a [HT_3] healthcare provider?
2. …been seen in the emergency room?
3. …spent one or more nights in the hospital?
4. …been screened for cancer? (mammogram, Pap test, colonoscopy, lung cancer)
5. …been treated for cancer?

[SPACE]

6. None of the above [SP] [ANCHOR]

[RANDOMIZE RESPONSE OPTIONS]

[MP]

Q3.

Deleted:

In the past 12 months, have <i><u>*any of your loved ones...*</u></i>

RESPONSE OPTIONS:

1. ...been seen in the emergency room?
2. ...spent one or more nights in the hospital?
3. ...been treated for cancer?

[SPACE]

4. None of the above [SP] [ANCHOR]

[SHOW Q4 AND Q5 ON THE SAME PAGE]

[SP]

Q4.

Have you ever been told by a [HT_3] healthcare provider that you have cancer?

RESPONSE OPTIONS:

1. Yes
2. No

[SP]

Q5.

Do you have a family history of cancer?

RESPONSE OPTIONS:

1. Yes
2. No

[SP]

Q6.

Would you say that in general your health is...

RESPONSE OPTIONS:

1. Poor
2. Fair
3. Good
4. Very Good
5. Excellent

[RANDOMIZE RESPONSE OPTIONS]

Deleted:

[SP]
Q8.
What kind of [HT_3] healthcare provider do you typically go if you are sick or need advice about your health??

RESPONSE OPTIONS:

1. A [HT_3] healthcare provider's office
2. A hospital clinic
3. Other clinic or health center
4. Urgent care
5. Emergency room
6. Other [TEXTBOX] [ANCHOR]
7. I don't have a regular healthcare provider

[SP]
Q9.
Approximately when was the last time you saw a [HT_3] healthcare provider?

RESPONSE OPTIONS:

1. Within the past year
2. Within the past 2 years
3. Within the past 5 years
4. More than 5 years ago
5. Have never seen a healthcare provider

[SP]
Q10.
Are you now covered by any form of health insurance or health plan (this includes Medicare, Medicaid, private health insurance and insurance plans available through healthcare.gov)?

RESPONSE OPTIONS:

1. Yes
2. No

[GRID; SP]
Q11.
For you, how true are the following statements?

GRID ITEMS [RANDOMIZE]:

A. [HT_2] The healthcare system in this country is easy to use

Deleted:

B. I can get the healthcare I need when I need it
C. I get all the information I need about my health from my [HT_3] healthcare provider
D. I could access my [HT_4] electronic health record if I wanted to
E. In general, I am satisfied with the treatment I receive from my [HT_3] healthcare providers

RESPONSE OPTIONS:

1. Not true
2. Somewhat true
3. Fairly true
4. Very true

[GRID; SP]
Q12.
For you, how true are the following statements?

GRID ITEMS [RANDOMIZE]:

A. Most healthcare systems in this country are too big to care about individual patients
B. [HT_1] My healthcare system is too big to care about me
C. Healthcare systems in this country work to prevent harm to their patients
D. I feel respected when I seek health care

RESPONSE OPTIONS:

1. Not true
2. Somewhat true
3. Fairly true
4. Very true

[GRID; SP]
Q13.
For you, how true are the following statements?

GRID ITEMS [RANDOMIZE]:

A. [HT_1] My healthcare system treats me fairly
B. [HT_1] My healthcare system treats me with kindness

Deleted:

RESPONSE OPTIONS:

1. Not true
2. Somewhat true
3. Fairly true
4. Very true

[SP]

Q14.

Have <u>you<u> ever experienced discrimination, or been hassled or made to feel inferior while getting medical care?

RESPONSE OPTIONS:

1. Yes
2. No

[SHOW IF Q14=1]

Q14AA.

[SP]

How often has this happened?

RESPONSE OPTIONS:

1. Once
2. 2 or 3 times
3. 4 or more times

[SHOW IF Q14=1]

Q14A.

[SP]

What do you think was the <u>main<u> reason for this experience?

RESPONSE OPTIONS:

1. Ancestry or national origin
2. Gender
3. Race
4. Age
5. Religion
6. Height
7. Weight

Deleted:

8. Shade of skin color
9. Sexual orientation
10. Education or income level
11. Physical disability
12. Speaking English as a second language
13. Other/ please specify [TEXTBOX]

Q15.

How often do you feel that racial/ethnic groups who are not white, such as African Americans and Latinos, are discriminated against in [HT_2] the healthcare system?

RESPONSE OPTIONS:

1. Never
2. Rarely
3. Sometimes
4. Often

[GRID; SP]

Q16.

For you, how true are the following statements?

GRID ITEMS [RANDOMIZE]:

A. I find ways to help others less fortunate than me
B. The dignity and well-being of all should be the most important concern in any society
C. One of the problems of today's society is that people are often not kind enough to others
D. All people who are unable to provide for their own needs should be helped by others

RESPONSE OPTIONS:

1. Not true
2. Somewhat true
3. Fairly true
4. Very true

[SP]

VIDEO_INT.

Deleted:

In the next section of the survey you will see a two-minute video that explains how [HT_5] health information is used and shared in [HT_2] the healthcare system. There will be questions that follow the video.

[SPACE]

<u>You will need to have your sound on while viewing this video. <u>

[SPACE]

Are you able to turn your sound on now?

RESPONSE OPTIONS:

1. Yes
2. No

[SHOW IF VIDEO_INT=1]

VIDEO1.

<u>Instructions for watching your video</u>:

- The continue button will appear once the video has ended.
- Do not fast forward through the video.
- Do not skip past the video before viewing it once.
- You may re-watch the video multiple times.
- The video is best viewed horizontally if watched on a mobile phone.
- Click on the image to start watching the video. Make sure you hear it.

[PLAY VIDEO HERE: Link to Video]

[DELAY PRESENTATION OF CONTINUE BUTTON FOR 120 SECONDS]

SHOW IF VIDEO_INT=1

VIDEO.

Were you able to <u>see<u> and <u>hear<u> the video?

RESPONSE OPTIONS:

1. Yes
2. No

SHOW IF VIDEO_INT=2,98 OR VIDEO=2,98

[DISPLAY]

TRANSCRIPT.

Today's technologies, from genome sequencing to [HT_4] electronic health records, are turning [HT_5] health information into a valuable resource for answering health questions and improving care.

[SPACE]

Deleted:

Imagine you have a friend, Florence, who was recently diagnosed with breast cancer. The handling of her [HT_5] health information looks very different today than it did 10 years ago. This will impact the care that she and other patients will receive.

[SPACE]

Florence's doctors will collect information about her health history, health behaviors, family history and maybe even her neighborhood and job. Doctors might also collect her genetic information using samples of her tumor as well as her normal blood cells to more precisely tailor or personalize her treatment. Blood left over from those tests might be set aside to be used for research. Using a team approach, dozens of people involved in her treatment may look at her chart to help support her care…

[SPACE]

[HT_5] Health information from patients like Florence also typically travels out to many other users in her health system who may not be involved directly in her care – insurers, billers, and analysts who could learn from the outcomes of her treatment.

[SPACE]

Precision health companies might collect and store archives of health data or use it to develop new drugs or digital tools for improving diagnosis and treatment. There are laws designed to protect Florence's privacy, but some of her information can still be shared after personal "identifiers" like her name and address are removed.

[SPACE]

With all of these users and uses of patients' data, Florence, and patients like her, are able to contribute to the improvement of their own medical care and the care of other patients like them. But these changes in the ways health care is conducted also come with some new questions for Florence and for all of us – questions about trust, privacy, duty, and the tradeoffs that come with sharing data.

[SPACE]

This survey asks you to reflect on what you think about the use and sharing of all of this health data. Thank you for your time!

[GRID; SP]

Q17.

Based on what you saw in the video or read in the transcript, are the following statements true or false?

GRID OPTIONS [RANDOMIZE]:

A. [HT_5] Health information can only be used for treating patients. (F)
B. Blood left over from tests used for treating or diagnosing a disease might be set aside to be used for research. (T)
C. Florence has cancer. (T)

RESPONSE OPTIONS:

1. True
2. False

[DISPLAY]
Now that you've heard Florence's story and seen how health information can be shared, we'd like to know what you think about the use and sharing of health information.
[SPACE]
The next questions are about the use of your health information for research.
[SPACE]
Your <u>health information</u> is information about you and your medical treatment history including diagnoses, medications, treatment plans, immunization dates, allergies, radiology images, and laboratory and test results.
[SPACE]
Your health information can be <i> "*de-identified*."</i> This means that "identifying information" about <i>*you*</i> <u>is</u> <i> *removed*</i> from your health information. Identifying information includes things like your name, address, date of birth, etc. De-identified information can then be given to researchers to study things like healthcare costs, quality, and diseases.
[SPACE]
<u>University researchers</u> are people who work for colleges or universities. These researchers might use health information to understand how people use [HT_2] the healthcare system, how [HT_3] healthcare providers treat patients, and a wide variety of other health related topics. University researchers might also use [HT_9] biospecimens for research on how illness works, which treatments are most effective, or how genetics affect illness. University researchers may or may not be connected to your hospital in some way.

[GRID; SP]

Q18.

For you, how true are the following statements?

GRID ITEMS [RANDOMIZE]:

A. I am comfortable with <u>[HT_8] university researchers</u> using my [HT_7] <i>*de-identified*</i> [HT_5] health information
B. I would like to be notified if <u>[HT_8] university researchers</u> will use my [HT_7] <i>*de-identified*</i> [HT_5] health information

RESPONSE OPTIONS:

1. Not true
2. Somewhat true
3. Fairly true
4. Very true

[GRID; SP]

Q19.

Your [HT_5] health information can be "<u>identified</u>." This means that [HT_10]"identifying information" about you <u>is linked to</u> your health information. Identifying information includes

things like your name, address, date of birth, etc. Identified information can then be given to researchers to study things like healthcare costs, quality, and diseases.
[SPACE]
For you, how true are the following statements?

GRID ITEMS [RANDOMIZE]:

A. I am comfortable with [HT_8] <u>university researchers</u> using my <i>*identified*</i> health information.
B. I would like to be notified if [HT_8] <u>university researchers</u> will use my <i>*identified*</i> health information.

RESPONSE OPTIONS:

1. Not true
2. Somewhat true
3. Fairly true
4. Very true

[GRID; SP]

Q20.

Your <u>biospecimens</u> may be collected during the course of your treatment. Biospecimens include blood from a blood test, or tissue or tumor samples from a biopsy. Your biospecimens contain your DNA. Sometimes when there are biospecimens left over from your healthcare (such as blood or urine left over from a diagnostic test) that otherwise would be thrown away, those leftover biospecimens might be used for research.
[SPACE]
Biospecimens can be "<u>[HT_7]de-identified</u>." This means that [HT_10] "identifying information" about you <u>is not linked to your</u> biospecimens. Identifying information includes things like your name, address, date of birth, etc. De-identified biospecimens can be given to researchers to study things like healthcare costs, healthcare quality, and diseases.
[SPACE]
For you, how true are the following statements?

GRID ITEMS [RANDOMIZE]:

A. I am comfortable with [HT_8] <u>university researchers<u> using my <i>*de-identified*</i> biospecimens.
B. I would like to be notified if [HT_8] <u>university researchers</u> use my <i>*de-identified*</i> biospecimens.

RESPONSE OPTIONS:

1. Not true
2. Somewhat true
3. Fairly true
4. Very true

Deleted:

[GRID; SP]

Q21.

[HT_9] Biospecimens can be "<u>identified</u>." This means that [HT_10] "identifying information" about you <u>is linked to</u> your biospecimens. Identifying information includes things like your name, address, date of birth, etc. Identified [HT_9] biospecimens can be given to researchers to study things like health care costs, quality, and diseases.
[SPACE]

For you, how true are the following statements?

GRID ITEMS [RANDOMIZE]:

A. I am comfortable with [HT_8] <u>university researchers<u> using my <i>*identified*</i> biospecimens.
B. I would like to be notified if [HT_8] <u>university researchers<u> use my <i>*identified*</i> biospecimens.

RESPONSE OPTIONS:

1. Not true
2. Somewhat true
3. Fairly true
4. Very true

[GRID; SP]

Q22.

The next questions are about the use of your [HT_5] health information by <u>quality analysts</u>.

[SPACE]

<u>Quality analysts<u> are people who work for hospitals or clinics. They use patient health information at their hospital or clinic to check on, and improve, how their organization is working. They often study the cost of healthcare, their organization's efficiency on things like waiting room times, and the health of patients at their hospital or clinic.

[SPACE]

For you, how true are the following statements about <u>health information<u>?

GRID ITEMS [RANDOMIZE]:

A. I am comfortable with quality analysts using my [HT_7] <i>*de-identified*</i> <u>health information</u>.
B. I would like to be notified if quality analysts use my [HT_7] <i>*de-identified*</i> <u>health information</u>.
C. I am comfortable with quality analysts using my [HT_10] <i>*identified*</i> <u>health information</u>.
D. I would like to be notified if quality analysts use my [HT_10] <i>*identified*</i> <u>health information</u>.

Deleted:

RESPONSE OPTIONS:
1. Not true
2. Somewhat true
3. Fairly true
4. Very true

[GRID; SP]

Q23.

For you, how true are the following statements about [HT_9] <u>biospecimens</u>?

GRID ITEMS [RANDOMIZE]:

A. I am comfortable with [HT_11] quality analysts using my [HT_7] <i>*de-identified*</i> <u>biospecimens</u>.
B. I would like to be notified if [HT_11] quality analysts use my [HT_7] <i>*de-identified*</i> <u>biospecimens</u>.
C. I am comfortable with [HT_11] quality analysts using my [HT_10] <i>*identified*</i> <u>biospecimens</u>.
D. I would like to be notified if [HT_11] quality analysts use my [HT_10] <i>*identified*</i> <u>biospecimens</u>.

RESPONSE OPTIONS:
1. Not true
2. Somewhat true
3. Fairly true
4. Very true

[GRID; SP;4,4]

Q24.

The next questions are about the use of your [HT_5] health information by <u>commercial companies</u>.

[SPACE]

<u>Commercial companies</u> are third-party companies that are not part of a hospital. For example, a third-party commercial company may conduct genetic tests and analyze information for a hospital or [HT_3] healthcare provider for a fee when a hospital is not be able to conduct the test on their own. Commercial companies may keep the information for their own use.

[SPACE]

For you, how true are the following statements?

GRID ITEMS [RANDOMIZE]:

A. I am comfortable with commercial companies using my [HT_7] <i>*de-identified*</i> <u>health information</u>.

B. I would like to be notified if commercial companies use my [HT_7] <i>*de-identified*</i> <u>health information</u>.
C. I am comfortable with commercial companies using my [HT_10] <i>*identified*</i> _<u>health information</u>.
D. I would like to be notified if commercial companies use my [HT_10] <i>*identified*</i> <u>health information</u>.
E. I am comfortable with commercial companies using my [HT_7] <i>*de-identified*</i> [HT_9] <u>biospecimens</u>.
F. I would like to be notified if commercial companies use my [HT_7] <i>*de-identified*</i> [HT_9] <u>biospecimens</u>.
G. I am comfortable with commercial companies using my [HT_10] <i>*identified*</i> [HT_9] <u>biospecimens</u>.
H. I would like to be notified if commercial companies use my [HT_10] <i>*identified*</i> [HT_9] <u>biospecimens</u>.

RESPONSE OPTIONS:

1. Not true
2. Somewhat true
3. Fairly true
4. Very true

[SP]

Q25.

How confident are you that [HT_7] de-identifying [HT_5] health information protects your privacy?

RESPONSE OPTIONS:

1. Not at all confident
2. Somewhat confident
3. Fairly confident
4. Very confident

[GRID; SP]

Q26.

How often would you like to be notified about the use of each of the following in research studies that start at your hospital and are shared with a [HT_12] commercial company?

GRID ITEMS [RANDOMIZE]:

A. Your genetic information or DNA
B. Your [HT_9] biospecimens
C. Your [HT_10] identified [HT_5] health information
D. Your [HT_7] de-identified [HT_5] health information

Deleted:

RESPONSE OPTIONS:

1. Never
2. Just once
3. Once every five years
4. Once a year
5. Every time I visit my [HT_3] healthcare provider
6. Every time the information is used

[GRID; SP]

Q27.

Suppose you are a cancer patient at a leading cancer center and your [HT_3] healthcare provider wants to use your DNA to see if you might be a good candidate for a particular cancer treatment. Your cancer center shares DNA and [HT_5] health information with third-party [HT_12] commercial companies when it is unable to perform the analysis themselves.

[SPACE]

How comfortable are you with a third-party commercial company…

GRID ITEMS [RANDOMIZE]:

A. …using your DNA and health information to improve the diagnosis and treatment of cancer in <u><i>*other*</i></u> patients
B. …developing predictions about how you will respond to a particular cancer treatment
C. …storing your DNA and health information
D. …sharing predictions about how you will respond to cancer treatment with insurance companies
E. …selling [HT_7] de-identified health information to pharmaceutical companies

RESPONSE OPTIONS:

1. Not comfortable
2. Somewhat comfortable
3. Fairly comfortable
4. Very comfortable

[GRID; SP]

Q28.

In the future, [HT_3] healthcare providers may be able to treat some diseases by making changes to patients' DNA, which is also called gene-editing. To make this possible, researchers need to use [HT_9] biospecimens (e.g. blood or tissue) donated from research participants.

[SPACE]

How comfortable are you with sharing <u>your own</u> [HT_7] <i>*de-identified*</i> biospecimen for research on gene-editing in the following ways?

GRID ITEMS [RANDOMIZE]:

A. To develop gene-editing methods that treat disease or disability.
B. To develop gene-editing methods that enhance a person physically (make them stronger or faster) or mentally (increase intelligence).

RESPONSE OPTIONS:

1. Not comfortable
2. Somewhat comfortable
3. Fairly comfortable
4. Very comfortable

[GRID; SP]

Q29.

In some cases, researchers would like to use [HT_10] <i>*identified*</i> [HT_9] biospecimens—samples linked to information that would identify you as the donor.

[SPACE]

How comfortable are you with sharing <u><u>your own</u></u> <i>*identified*</i> biospecimens for research on gene-editing in the following ways?

GRID ITEMS [RANDOMIZE]:

A. To develop gene-editing methods that treat disease or disability.
B. To develop gene-editing methods that enhance a person physically (make them stronger or faster) or mentally (increase intelligence).

RESPONSE OPTIONS:

1. Not comfortable
2. Somewhat comfortable
3. Fairly comfortable
4. Very comfortable

[SP]

Q30.

In the U.S., the federal government is a major funder of medical research using tax revenue.
[SPACE]
How comfortable are you with your tax dollars being used to support research on gene-editing?

RESPONSE OPTIONS:

1. Not comfortable
2. Somewhat comfortable
3. Fairly comfortable
4. Very comfortable

Deleted:

[GRID; SP]

Q31.

How optimistic are you that gene-editing will have a <u>positive</u> impact:

GRID ITEMS [RANDOMIZE]:

A. On you
B. On your family
C. On society

RESPONSE OPTIONS:

1. Not at all optimistic
2. Somewhat optimistic
3. Quite optimistic
4. Very optimistic

[GRID; SP]

Q32.

How fearful are you that gene-editing will have a <u>negative</u> impact:

GRID ITEMS [RANDOMIZE]:

A. On you
B. On your family
C. On society

RESPONSE OPTIONS:

1. Not at all fearful
2. Somewhat fearful
3. Quite fearful
4. Very fearful

[GRID; SP]

Q33.

The next questions ask for your opinions about whether people should share their [HT_5] health information in general.

[SPACE]

For you, how true are the following statements?

GRID ITEMS [RANDOMIZE]:

A. People have an ethical obligation to allow their health information to be used for <u>healthcare quality analysis</u>

B. People have an ethical obligation to allow their health information to be used for <u>research</u>

RESPONSE OPTIONS:

1. Not true
2. Somewhat true
3. Fairly true
4. Very true

[GRID; SP]

Q34.

For you, how true are the following statements about the organizations that have your [HT_5] health information and share it? Organizations include groups such as [HT_3] healthcare providers' offices, hospitals, insurance companies, and [HT_8] university researchers. (If you are unsure, please make your best guess.)

[SPACE]

<i>*The organizations that have my health information and share it...*</i>

GRID ITEMS [RANDOMIZE]:

A. Try hard to be fair in dealing with others
B. Would try to hide a serious mistake they made
C. Tell me how my health information is used
D. Would never mislead me about how my health information is used
E. Have a particular interest in collecting my [HT_9] biospecimens compared to other people's

RESPONSE OPTIONS:

1. Not true
2. Somewhat true
3. Fairly true
4. Very true

[GRID; SP]

Q35.

<i>*The organizations that have my [HT_5] health information and share it...*</i>

GRID ITEMS [RANDOMIZE]:

A. Are not good at their jobs
B. Have specialized capabilities that can promote innovation in health

Deleted:

C. Can use large amounts of data to improve patient care

RESPONSE OPTIONS:

1. Not true
2. Somewhat true
3. Fairly true
4. Very true

[GRID; SP]

Q36.

<i>*The organizations that have my* [HT_5] *health information and share it...*</i>

GRID ITEMS [RANDOMIZE]:

C. Can be trusted to use my health information responsibly
D. Think about what is best for me
E. Act in an ethical manner

RESPONSE OPTIONS:

1. Not true
2. Somewhat true
3. Fairly true
4. Very true

[GRID; SP]

Q37.

<i>*The organizations that have my* [HT_5] *health information and share it...*</i>

GRID ITEM [RANDOMIZE]:

A. Deliberately withhold important information from me about my medical care
B. Disclose their financial conflicts of interest
C. Experiment on patients without telling them
D. Treat everyone the same, regardless of their race or ethnicity
E. Treat everyone the same, regardless of their income

RESPONSE OPTIONS:

1. Not true
2. Somewhat true

Deleted:

3. Fairly true
4. Very true

[GRID; SP]

Q38.

For you, how true are the following statements about [HT_3] <u><i>*health care providers*</i></u>?

GRID ITEMS [RANDOMIZE]:

A. Health care providers care most about making money for themselves
B. Health care providers do not care about helping people like me
C. I trust health care providers to use my [HT_5] health information responsibly
D. Health care providers disclose their conflicts of interest
E. All things considered, health care providers in this country can be trusted

RESPONSE OPTIONS:

1. Not true
2. Somewhat true
3. Fairly true
4. Very true

[GRID; SP]

Q39.

For you, how true are the following statements?

GRID ITEMS [RANDOMIZE]:

A. The privacy policies of [HT_1] my healthcare system are clear to me
B. I am satisfied with the level of access I have to information in my [HT_4] electronic health record
C. I am confident in my ability to manage how my [HT_5] health information is used
D. [HT_1] My healthcare system respects my privacy

RESPONSE OPTIONS:

1. Not true
2. Somewhat true
3. Fairly true
4. Very true

[GRID; SP]

Deleted:

Q40.

For you, how true are the following statements?

GRID ITEMS [RANDOMIZE]:

A. I should have more control over how my [HT_5] health information is used
B. [HT_1] My healthcare system is transparent about how my [HT_5] health information is used
C. [HT_1] My healthcare system would notify me if the security of my [HT_5] health information had been compromised

RESPONSE OPTIONS:

1. Not true
2. Somewhat true
3. Fairly true
4. Very true

[GRID; SP]

Q41.

For you, how true are the following statements?

GRID ITEMS [RANDOMIZE]:

B. I worry that private information about my health could be used against me
C. I worry my [HT_5] health information is available to people who have no business seeing it
D. There are some things I would not tell my [HT_3] healthcare providers because I can't trust them with the information
E. [HT_1] My healthcare system discloses its conflicts of interest

RESPONSE OPTIONS:

1. Not true
2. Somewhat true
3. Fairly true
4. Very true

[SP]

Q42.

Have you ever kept information from your [HT_3] healthcare provider because you were concerned about privacy or security?

RESPONSE OPTIONS:

1. Yes
2. No

Deleted:

[GRID;3,3; SP]

Q43.

What do you think of the following statements: true or false?

GRID ITEMS [RANDOMIZE]:

A. My [HT_3] healthcare provider is the only person who decides how information in my [HT_4] electronic health record is used
B. Health insurance companies are prohibited from using my [HT_5] health information to deny me coverage
C. I own my [HT_5] health information
D. My permission is required for research using my [HT_7] de-identified [HT_5] health information
E. My genetic code is specific to me
F. Health organizations are required to report large-scale data breaches

RESPONSE OPTIONS:

1. True
2. False
77. I don’t know

[GRID; SP]

Q44.

For you, how true are the following statements?

GRID ITEMS [RANDOMIZE]:

A. [HT_1] My healthcare system will use my [HT_5] health information how they see fit, regardless of my preferences
B. Healthcare systems in this country respect patients’ opinions about how their [HT_5] health information should be used

RESPONSE OPTIONS:

1. Not true
2. Somewhat true
3. Fairly true
4. Very true

[GRID; SP]

Deleted:

Q45.

The next questions ask about your perceptions of the uses of [HT_5] health information and the policies that are in place to protect it.

[SPACE]

For you, how true are the following statements?

GRID ITEMS [RANDOMIZE]:

A. I should have a right to participate in medical research
B. Information about my health should be kept indefinitely in my [HT_4] electronic health record
C. I should be able to delete my [HT_4] electronic health record
D. I should be able to access all of my [HT_3] healthcare providers through a single patient portal

RESPONSE OPTIONS:

1. Not true
2. Somewhat true
3. Fairly true
4. Very true

[GRID; SP]

Q46.

For you, how true are the following statements?

GRID ITEMS [RANDOMIZE]:

A. It is important that I know who has [HT_5] health information about me
B. I should be able to find out how my [HT_5] health information has been shared

RESPONSE OPTIONS:

1. Not true
2. Somewhat true
3. Fairly true
4. Very true

[GRID; SP]

Q47.

For you, how true are the following statements?

GRID ITEMS [RANDOMIZE]:

A. It is okay for law enforcement to access [HT_5] health information
C. Existing laws provide a reasonable level of protection for the privacy of patient information
D. I am confident that electronic [HT_5] health information is sufficiently protected by current law and regulation

RESPONSE OPTIONS:

1. Not true
2. Somewhat true
3. Fairly true
4. Very true

[GRID;3,3; SP]

Q48.

Suppose [HT_1] your healthcare system charges a fee to third-party [HT_12] commercial companies that want to keep [HT_9] biospecimens left over after testing.   This makes money for the hospital.

[SPACE]

For you, how true are the following statements?

[SPACE]

If my healthcare system makes money from my biospecimens…

GRID ITEMS [RANDOMIZE]:

A. …they should use the money to provide health care for other people who can't afford it.
B. …they should use the money to provide health care for other people who have the same health problems as me.
C. …they should use the money to improve quality of care in my healthcare system.
D. …they should use the money to support future research on my health problems.
E. …they should use the money to support future research on any kind of health problem.
F. …they should use it however they want.

RESPONSE OPTIONS:

1. Not true
2. Somewhat true
3. Fairly true
4. Very true

[SP]

Q49.

Some companies like 23andMe and AncestryDNA test people's genetic material from saliva to trace their ancestry.

Deleted:

[SPACE]

Given what you know about companies like 23andMe and AncestryDNA, do you generally have a favorable or unfavorable opinion of these companies?

RESPONSE OPTIONS:

1. Very favorable
2. Somewhat favorable
3. Somewhat unfavorable
4. Very unfavorable

[SP]

Q50.

Are you comfortable with <u>drug companies</u> purchasing genetic and ancestry data from companies like 23andMe or AncestryDNA?

RESPONSE OPTIONS:

1. Yes
2. No

[SP]

Q51.

Are you comfortable with <u>law enforcement</u> using genetic and ancestry data from companies like 23andMe or AncestryDNA?

RESPONSE OPTIONS:

1. Yes
2. No

[SP]

Q53.

Have you experienced problems with stolen or misused personal information (e.g., social security number, credit or debit cards, and bank accounts) within the last five years? For example, have you spent time clearing up credit accounts or your credit report because someone stole your personal information?

RESPONSE OPTIONS:

1. Yes, I am currently experiencing problems
2. Yes, but all problems have been resolved

Deleted:

3. No, I have not experienced any problems within the past five years

---

[SP]
Q54.
I believe my financial information has been compromised as the result of a data breach or hacking.

RESPONSE OPTIONS:
1. Yes
2. No

[GRID; SP]
Q55.
How concerned are you about the following recent events:

GRID ITEMS [RANDOMIZE]:
A. Facebook sharing information with Cambridge Analytica for political purposes
B. Data breach of people's social security numbers and driver's license numbers at Equifax
C. Sloan Kettering hospital executives using hospital data for their own startup company
D. Marriott data breach of passport numbers and credit card numbers

RESPONSE OPTIONS:
1. Not at all concerned
2. Somewhat concerned
3. Fairly concerned
4. Very concerned

[SHOW IF MISSING P_PARTYID7]
[SP]
PID1.
Do you consider yourself a Democrat, a Republican, an independent or none of these?

RESPONSE OPTIONS:
1. Democrat
2. Republican
3. Independent
4. None of these

Deleted:

Previously programmed as PIDA from TESS 007
[SHOW IF PID1=1]
[SP]
PIDA.
Do you consider yourself a strong or moderate Democrat?

RESPONSE OPTIONS:
1. Strong Democrat
2. Moderate Democrat

Previously programmed as PIDB from TESS 007
[SHOW IF PID1=2]
[SP]
PIDB.
Do you consider yourself a strong or moderate Republican?

RESPONSE OPTIONS:
1. Strong Republican
2. Moderate Republican

Previously programmed as PIDi from TESS 007
[SHOW IF PID1=3, 4, 77, 98, 99]
[SP]
PIDi.
Do you lean more toward the Democrats or the Republicans?

RESPONSE OPTIONS:
1. Lean Democrat
2. Lean Republican
3. Don’t lean

[DOUBLE PROMPT IF REFUSED]

[NUMBERBOX; RANGE 1-20]

HHSIZE2.

<u>Including yourself</u>, how many people live in your household?

[NUMBERBOX; RANGE 0-999,000]
[PROMPT TWICE IF REFUSED WITH CUSTOM MESSAGE “We know that questions about income are sensitive, and understand if you would not like to answer this question. However, some of our key research questions require us to have this information for respondents of this survey.”
INCOME2.
What was your total <u>household,/u. income in 2018?

Deleted:

COMPUTE DOV_FPL BASED ON THE CHART BELOW. IF INCOME2 IS AT OR BELOW A GIVEN VALUE FOR A SPECFIC HHSIZE2 DOV_FPL=1. NOTICE THERE ARE DIFFERENT INCOME2 CRITERIA DEPENDING ON S_STATE VALUES AS INDICATED BY THE 3 INCOME2 COLUMNS

HHSIZE2	INCOME2 FOR S_STATE NE AK (Alaska) or HI (Hawaii)	INCOME2 FOR S_STATE= AK (Alaska)	INCOME2 FOR S_STATE= HI (Hawaii)
1	$24,980	$31,200	$28,760
2	33,820	42,260	38,920
3	42,660	53,320	51,080
4	51,500	64,380	59,240
5	60,340	75,440	69,400
6	69,180	86,500	79,560
7	78,020	97,560	89,720
8	86,860	108,620	99,880
9	95,700	119,680	110,040
10	104,540	130,740	120,200
11	113,380	141,800	130,360
12	122,220	152,860	140,520
13	131,060	163,920	150,680
14	139,900	174,980	160,840
15	148,740	186,040	171,000
16	157,580	197,100	181,160
17	166,420	208,160	191,320
18	175,260	219,220	201,480
19	184,100	230,280	211,640
20	192,940	241,340	221,800

[TEXTBOX]

CLOSE1.

In thinking about [HT_5] health information sharing, do you have any comments you would like to share?

[MEDIUM TEXTBOX]

PM PLEASE ALWAYS HAVE THIS AND THE FOLLOWING LOGIC FOLLOW THE FINAL SUBSTANTIVE QUESTION OF THE SURVEY, AHEAD OF QFINAL

INSERT ITEM TIMESTAMPS: TIME_END, DATE_END

Deleted:

COMPUTE **TEST_TIME**
TEST_TIME = TIME_END – TIME_START

COMPUTE **TEST_DATE** = DATE_END

DISPLAY TESTING-ONLY SCREEN WITH VALUE FOR **TEST_TIME** & **TEST_DATE**

RE-COMPUTE QUAL=1 "COMPLETE"

SET CO_DATE, CO_TIME, CO_TIMER VALUES HERE

CREATE MODE_END
1=CATI
2=CAWI

SCRIPTING NOTES: PUT QFINAL1, QFINAL2, QFINAL3 in the same screen.
[SINGLE CHOICE]
QFINAL1.
Thank you for your time today. To help us improve the experience of AmeriSpeak members like yourself, please give us feedback on this survey.

[RED TEXT – CAWI ONLY] If you do not have any feedback for us today, please click "Continue" through to the end of the survey so we can make sure your opinions are counted and for you to receive your AmeriPoints reward.

Please rate this survey overall from 1 to 7 where 1 is Poor and 7 is Excellent.

Poor						Excellent
1	2	3	4	5	6	7

[SINGLE CHOICE – CAWI ONLY]
QFINAL2.
Did you experience any technical issues in completing this survey?

1. Yes – please tell us more in the next question
2. No

[TEXT BOX] [CATI version needs "no" option]
QFINAL3.
Do you have any general comments or feedback on this survey you would like to share? If you would like a response from us, please email support@AmeriSpeak.org or call (888) 326-9424.

[DISPLAY]

Deleted:

END.
[CATI version]
Those are all the questions we have. You have earned a reward of [INCENTWCOMMA] AmeriPoints for completing the survey. If you have any questions at all for us, you can email us at support@AmeriSpeak.org or call us toll-free at **888-326-942**4. Let me repeat that again: email us at support@AmeriSpeak.org or call us at **888-326-9424**. Thank you for participating in our new AmeriSpeak survey!

[CAWI version]
Those are all the questions we have. You have earned a reward of [INCENTWCOMMA] AmeriPoints for completing the survey. If you have any questions at all for us, you can email us at support@AmeriSpeak.org or call us toll-free at **888-326-9424**. Thank you for participating in our new AmeriSpeak survey!

You can close your browser window now if you wish or click Continue below to be redirected to the AmeriSpeak member website.
