# Supplementary material for: Do people have an ethical obligation to share their health information? Comparing narratives of altruism and health information sharing in a nationally representative sample
Source: PLoS One. 2020 Dec 31;15(12):e0244767. doi: 10.1371/journal.pone.0244767 (PMC7774955; doi:10.1371/journal.pone.0244767)
Supplement: Attachment — Submitted filename: Response to Reviewers.docx [file pone.0244767.s005.docx]

Dear Dr. Bhargava,

Thank you for considering our manuscript for publication and for allowing us to submit a revision. We also thank the reviewers for their supportive and helpful comments. We have included responses to comments below and all changes are included in the manuscript in tracked changes. In addition, we have made the data for this analysis available and have provided a data dictionary as Supporting Information Files.

Thank you.

| Editor comments | Authors’ response |
| --- | --- |
| 1. One reviewer has accepted the manuscript with few reservations about the depth and possible conclusions. You may consider including a section on limitations, implications and recommendations. Another reviewer has suggested minor edits. | We have extended the Limitations section to include some of the concerns Reviewer 1 brought up, on page 24:  *Third, our study draws exclusively on cross-sectional survey data limiting our ability to make causal inferences or understand the role of participants’ motivations and concerns more deeply. Future studies using qualitative and quantitative approaches would provide this critical insight.*  We provide several recommendations from pages 22-24; however, on page 23 we have added:  *This type of data can help us to develop policies and practices that address participants’ concerns while also tailoring policies to consider the intersection of lived experiences, concerned and preferences, and technological advancement.* |
| Reviewer #1: This is a piece within the complex area of consent and research participation that explores the motivation of research participants and the response to sharing health data for research, even as our health data is less secure than we imagine, and it is not possible to address our common health concerns using data analysis, AI and computation in a world of information networking and meshing. As there is no qualitative element, it is hard to explore the motivations and concerns in more detail. However this initial study, while bearing limitations in scope and application, can be a springboard for further deeper exploration and research in this space. Since there were many demographic variables, (possibly mirroring the population under study) it would be hard to arrive at information that can effectively inform policy. The depth of understanding is missing and the cross analysis leaves one in a further quandary, which one hopes will be the basis of a further, and better, study that would better elicit these perceptions. Hybrid studies are on way to improve understanding of the data collected. | Thank you for your supportive comments on our manuscript. We agree that there is a need to explore this complex area further using other approaches (e.g., qualitative methods) to capture some of the complexity underlying our quantitative findings. Our findings also guide further analyses focusing on specific variables that may be particularly relevant for policy. |
| Reviewer #2: The topic is interesting and the sample size is good. However, the language needs improvement. The presentation of tables could be in line with the journal guidelines. Please go through the attachment for an additional comment. | Thank you for your suggestions. We have made the following changes:  - On page 4, deleted “that research”  - On page 7, edited to “looking at”  - We are not sure what aspect of the tables is not aligned with journal guidelines. However, we have included gridlines and ensured that text justification is consistent. Our preference is to have the first column (variable names) right-justified, and subsequent columns (data) left-justified. |
| Wouldn’t confidentiality be threatened in such a case? Would the participants become more vulnerable? Will this lead them to participate to be viewed as positive to the idea? | Your point about people who might participate because they want to be viewed positively is particularly interesting. On page 24, we have added:  *These types of changes would require consideration of confidentiality (i.e., ensuring that the participant’s personal information is not shared with those who have benefited and vice versa) as well as robust informed consent processes to ensure that participants do not feel coerced or compelled to participate, for instance, for desirability.* |
| Journal requirements |  |
| 1. Please ensure that your manuscript meets PLOS ONE's style requirements, including those for file naming. | We confirm that our file names for the revision meet PLOS ONE’s style requirements. |
| 2. Please include additional information regarding the survey or questionnaire used in the study and ensure that you have provided sufficient details that others could replicate the analyses. For instance, if you developed a questionnaire as part of this study and it is not under a copyright more restrictive than CC-BY, please include a copy, in both the original language and English, as Supporting Information. | We have attached the whole survey as Supporting Information. |
| 3. We note that you have indicated that data from this study are available upon request. PLOS only allows data to be available upon request if there are legal or ethical restrictions on sharing data publicly. For information on unacceptable data access restrictions, please see <http://journals.plos.org/plosone/s/data-availability#loc-unacceptable-data-access-restrictions>. | We have included the data relevant for this analysis and a data dictionary as Supporting Information. |
